# Insights into Transport Function of the Murine Organic Anion-Transporting Polypeptide OATP1B2 by Comparison with Its Rat and Human Orthologues

**DOI:** 10.3390/toxics14010010

**Published:** 2025-12-20

**Authors:** Saskia Floerl, Annett Kuehne, Yohannes Hagos

**Affiliations:** PortaCellTec Biosciences GmbH, Science Park Va, Marie-Curie-Straße 8, 37079 Göttingen, Germany; floerl@portacelltec.de (S.F.); kuehne@portacelltec.de (A.K.)

**Keywords:** mOATP1B2, rOATP1B2, hOATP1B1, hOATP1B3, SLCO1B1, SLCO1B3, species differences, drugs, cyclosporine A, rifampicin, ritonavir, rosuvastatin, ketoconazole, olmesartan, odevixibat, amprenavir, clotrimazole

## Abstract

Organic anion-transporting polypeptides (OATPs) are key transporters of hepatic uptake for endogenous compounds and xenobiotics. Human OATP1B1 and OATP1B3 are well-studied due to their role in drug–drug interactions. In contrast, data on murine OATP1B2, the rodent orthologue of these transporters, are limited, despite its importance in early drug development. Here, we systematically compared the transport characteristics of mouse and rat OATP1B2 under identical experimental conditions. The K_m_ values for estrone-3-sulfate (E_1_S) and taurocholate (TCA) were 242 and 73 µM for mOATP1B2 and 90 and 16 µM for rOATP1B2. Nine clinically relevant drugs were evaluated for inhibitory effects, showing strong correlation between species. Cyclosporine A, ritonavir, odevixibat, rosuvastatin, and rifampicin markedly inhibited uptake. Rifampicin demonstrated species-specific differences, with higher IC_50_ values for mOATP1B2 (E_1_S: 9.6 µM; TCA: 7.7 µM) than rOATP1B2 (E_1_S: 1.1 µM; TCA: 2.4 µM). A comparison of the rodent data with the human orthologues revealed similar inhibition patterns but distinct substrate selectivity: hOATP1B1 showed high affinity for E_1_S but negligible TCA uptake, while hOATP1B3 transported TCA weakly but not E_1_S. This study provides insights into species-specific differences in OATP-mediated hepatic uptake and is therefore valuable for the interpretation of preclinical studies and their transfer to human pharmacology.

## 1. Introduction

The plasma membrane of cells contains a variety of transport proteins responsible for the uptake and efflux of hydrophilic substances. Organic anion-transporting polypeptides (OATPs) in humans and their orthologues in rodents mediate the cellular uptake of numerous endogenous compounds, as well as a wide range of pharmaceuticals and other xenobiotics. In the liver, human and rodent OATPs play a pivotal role in the import of substances into hepatocytes.

The murine organic anion-transporting polypeptide 1B2 (mOATP1B2) belongs to the solute carrier (SLC) family SLCO and is highly expressed in the liver [1,2]. It serves as the murine orthologue of the human organic anion-transporting polypeptides OATP1B1 and OATP1B3, as well as the rat OATP1B2 (rOATP1B2) [3]. The human transporters OATP1B1 and OATP1B3 are of substantial pharmaceutical and clinical relevance, as they are recognized as key sites for drug–drug interactions (DDIs). Consequently, these transporters are considered clinically significant and are routinely tested by the pharmaceutical industry for interactions with new molecular entities (NMEs), in accordance with recommendations from regulatory agencies such as the FDA and EMA [4,5].

Rodents, particularly mice and rats, are frequently employed in early-phase in vivo studies to assess the toxicity, pharmacokinetics, and clearance of drugs. Data derived from rodent models are essential for understanding the effects of drugs within the body and for identifying potential DDIs. However, not all findings from rodent models can be directly translated to humans, particularly in the context of transporter interactions. This limitation is illustrated by the fact that, in the OATP1B subfamily, mice and rats each possess only a single OATP1B2 protein, while humans express two orthologous transporters, OATP1B1 and OATP1B3 [6]. The human OATP1B1 and OATP1B3 genes likely underwent duplication during evolutionary history, resulting in transporters that share approximately 80% amino acid identity [2,7]. The murine OATP1B2 shares 81% amino acid identity with rat OATP1B2 and 64% and 66% identity with human OATP1B1 and OATP1B3, respectively (Clustal Omega, version 1.2.4). The full-length mOATP1B2 and rOATP1B2 consist of 689 and 678 amino acids, while the human OATP1B1 and OATP1B3 proteins consist of 691 and 702 amino acids, respectively. Mouse OATP1B2 is predominantly expressed in the liver and kidney, with lower expression level detected in the stomach, as demonstrated both at the mRNA and protein levels [2].

Despite the clinical importance of its human orthologues, the transport characteristics of mOATP1B2 remain relatively underexplored. Nevertheless, several known substrates of mOATP1B2 have been identified, including taurocholate, estrone-3-sulfate, estradiol 17β-glucuronide, pravastatin, atorvastatin, and rifampicin [2,7]. More extensive data are available regarding the interaction of substances with rat OATP1B2. Substrates of rOATP1B2 include taurocholate, BSP, estrone-3-sulfate, estradiol 17β-glucuronide, rosuvastatin, and olmesartan [8,9]. In addition, rifampicin, digoxin, and glyburide have been identified as inhibitors of rOATP1B2 [9].

The present study aims to systematically characterize the transport properties of murine OATP1B2, including uptake and inhibition assays with clinically relevant drugs known to interact with human OATP1B1 and OATP1B3. These findings are then compared to the corresponding data for the rat orthologue. Subsequently, the results from the rodent models are associated with data on human OATP1B1 and OATP1B3 to better understand the degree to which rodent transporter data can be extrapolated to humans.

## 2. Results

### 2.1. Transport Activity of Mouse and Rat OATP1B2 in Overexpressing HEK-293 Cells

**Figure 1:**
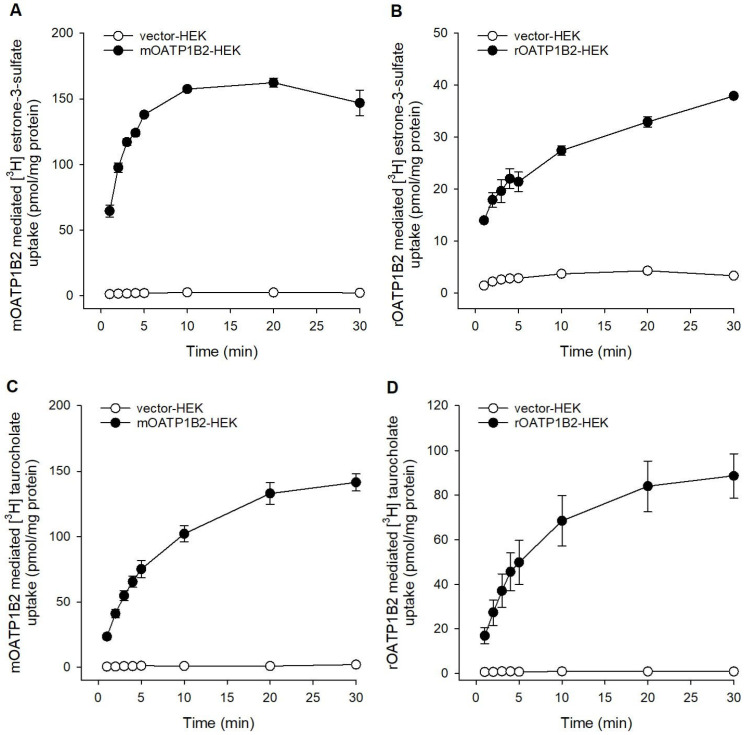
Uptake over time. Substrate uptake for [^3^H]-E_1_S and [^3^H]-TCA (1 µM each) was analyzed in (**A**,**C**) mOATP1B2—or (**B**,**D**) rOATP1B2-overexpressing HEK293 cells (closed symbols) and empty vector-transfected control HEK293 cells (open symbols) at incubation times of 1, 2, 3, 4, 5, 10, 20, and 30 min. Data are presented as means ± average deviation of triplicate determinations from two independent experiments.

To characterize the transport activity of mOATP1B2 in comparison to rOATP1B2, initially, substrate accumulation over time (1–30 min) was examined in overexpressing HEK293 cells with two different endogenous substrates (Figure 1). Estrone-3-sulfate (E_1_S) as well as taurocholate (TCA) uptake increased linearly up to 4 min in mOATP1B2 and rOATP1B2 cells, respectively. After 20 min the uptake of TCA facilitated by mOATP1B2 and rOATP1B2 was at a steady state, while the steady-state level of E_1_S started at 10 min for mOATP1B2 and was not reached completely for rOATP1B2 after 30 min. Further E_1_S and TCA uptake experiments were performed for both transporters within initial and linear time ranges. At the initial time of 1 min, the uptake ratios for mOATP1B2-HEK cells versus vector-HEK cells were more than 50-fold of E_1_S as well as TCA. In comparison, the uptake ratios for rOATP1B2 were around 10-fold for E_1_S and around 20-fold for TCA.

**Figure 2:**
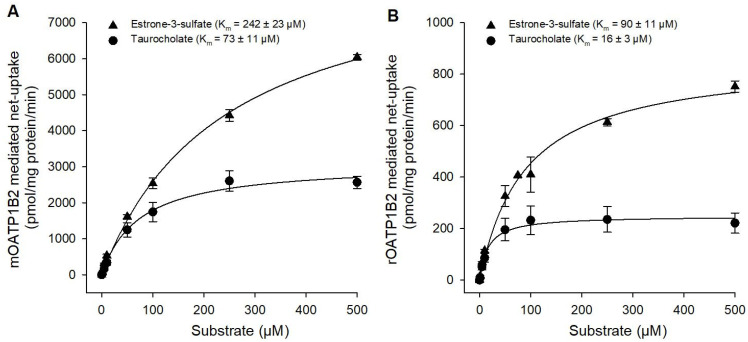
Concentration-dependent uptake. Kinetics of (**A**) mOATP1B2- and (**B**) rOATP1B2-mediated uptake of [^3^H]-E_1_S and [^3^H]-TCA, incubated 1 min at 37 °C with increasing concentrations of unlabeled substrate (containing 10 nM radiolabeled substrate, respectively). Net uptake (mean ± average deviation of two independent experiments) was fitted to the Michaelis–Menten equation using nonlinear regression to determine the affinity constant (K_m_) with Sigma Plot version 13.0.

Subsequently, the concentration-dependent E_1_S and TCA uptake was measured to assess the affinities of mOATP1B2 and rOATP1B2 for these substrates (Figure 2). Therefore, we measured the uptake of E_1_S or TCA in a transport buffer containing 10 nM ^3^H-labeled substrate in the presence of increasing concentrations of non-labeled substrate. The uptake of E_1_S revealed a K_m_ of 242 ± 23 µM for mOATP1B2 (V_max_ 8881 ± 390 pmol/mg protein/min) and a K_m_ of 90 ± 11 µM for rOATP1B2 (V_max_ 859 ± 36 pmol/mg protein/min). The uptake of TCA showed K_m_ values of 73 ± 11 µM for mOATP1B2 (V_max_ 3095 ± 146 pmol/mg protein/min) and of 16 ± 3 µM for rOATP1B2 (V_max_ 249 ± 9 pmol/mg protein/min). In summary, both E_1_S and TCA are appropriate substrates for further experiments.

### 2.2. Interaction of Drugs with Mouse OATP1B2 Compared to Rat OATP1B2

**Figure 3:**
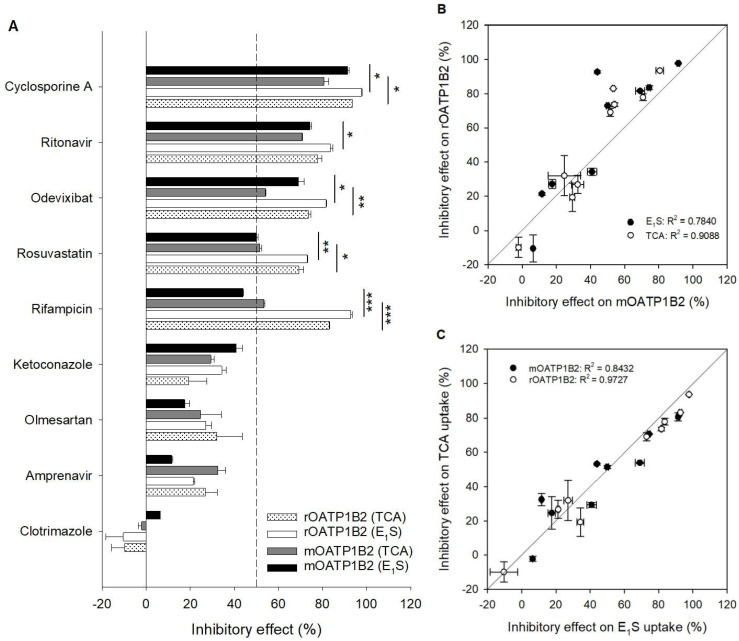
Inhibition of mOATP1B2- and rOATP1B2-mediated substrate uptake. (**A**) Inhibitory effects of various drugs (10 µM) on mOATP1B2- and rOATP1B2-mediated [^3^H]-E_1_S and [^3^H]-TCA uptake in transfected HEK cells (mean ± average deviation of two independent experiments, each in triplicate). For inhibition experiments vector- and transporter-transfected HEK293 cells were incubated for 1 min with 1 µM [^3^H]-E_1_S or [^3^H]-TCA (each containing 10 nM radiolabeled substrate). (**B**) Scatterplot analysis correlating inhibitory effects on mOATP1B2 and rOATP1B2 and (**C**) correlating inhibitory effects on substrates E_1_S and TCA. The lines indicate a perfect correlation. Statistical significance was assessed using a Student’s *t*-test. The level of significance is denoted by asterisks: one asterisk (*) indicates *p* ≤ 0.05, two asterisks (**) indicate *p* ≤ 0.01, and three asterisks (***) indicate *p* ≤ 0.001.

After demonstrating the substrate properties of [^3^H]-E_1_S and [^3^H]-TCA in the time- and concentration-dependent experiments, nine drugs from different drug classes were tested with both substrates with the intention to find suitable inhibitors for mOATP1B2 (Figure 3A) and compare the results with its orthologues.

An inhibition study was performed at a concentration of 1 µM for each substrate, which is well below the K_m_ of the transporters for these substrates. Here, mainly drugs were used that are known inhibitors for the orthologous transporters hOATP1B1, hOATP1B3, or rOATP1B2. So, the immunosuppressant cyclosporine A as well as the antibiotic rifampicin and the antiviral drugs ritonavir and amprenavir are known inhibitors of hOATP1B1 and hOATP1B3 [10,11]. The angiotensin II receptor antagonist olmesartan and the HMG-CoA reductase inhibitor rosuvastatin were described as substrates of hOATP1B1 and hOATP1B3 [12,13], and olmesartan was described as a substrate of rOATP1B2 [9]. Furthermore, the antifungal medications ketoconazole and clotrimazole, as well as the ASBT-inhibitor odevixibat, were included in this inhibition study.

The following results were obtained for these drugs regarding their inhibitory effect on mOATP1B2-E_1_S and TCA uptake: Cyclosporine A (91/81%) > ritonavir (74/71%) > rifampicin 44/53%) > odevixibat (69/54%) > rosuvastatin (50/52%) > ketoconazole (41/29%) > olmesartan (18/25%) > amprenavir (12/32%) > clotrimazole (6/−2%). The inhibition of rOATP1B2 E_1_S and TCA uptake resulted in the following inhibitory effects: Cyclosporine A (98/94%) > rifampicin 93/83%) > ritonavir (84/78%) > odevixibat (82/74%) > rosuvastatin (73/69%) > ketoconazole (34/19%) > olmesartan (27/32%) > amprenavir (21/27%) > clotrimazole (−10/−10%). In summary, five of the nine drugs showed inhibitory effects of 50% or more on mOATP1B2 and rOATP1B2 substrate uptake at an inhibitor concentration of 10 µM. The three drugs ketoconazole, olmesartan, and amprenavir showed inhibitory effects below 50% for both rodent transporters, and clotrimazole showed no inhibitory properties. A regression analysis (Figure 3B) showed a high correlation between the OATP1B2 of the two species: for [^3^H]-TCA the R^2^ was 0.9088 and for [^3^H]-E_1_S the R^2^ was 0.7840.

When comparing the inhibitory effects of the drugs on the [^3^H]-TCA and [^3^H]- E_1_S uptake, a correlation analysis (Figure 3C) showed for rOATP1B2 an R^2^ of 0.9727 and for mOATP1B2 an R^2^ of 0.8432.

### 2.3. Concentration-Dependent Inhibitory Effects of Cyclosporine A and Rifampicin on Mouse OATP1B2 and Rat OATP1B2

**Figure 4:**
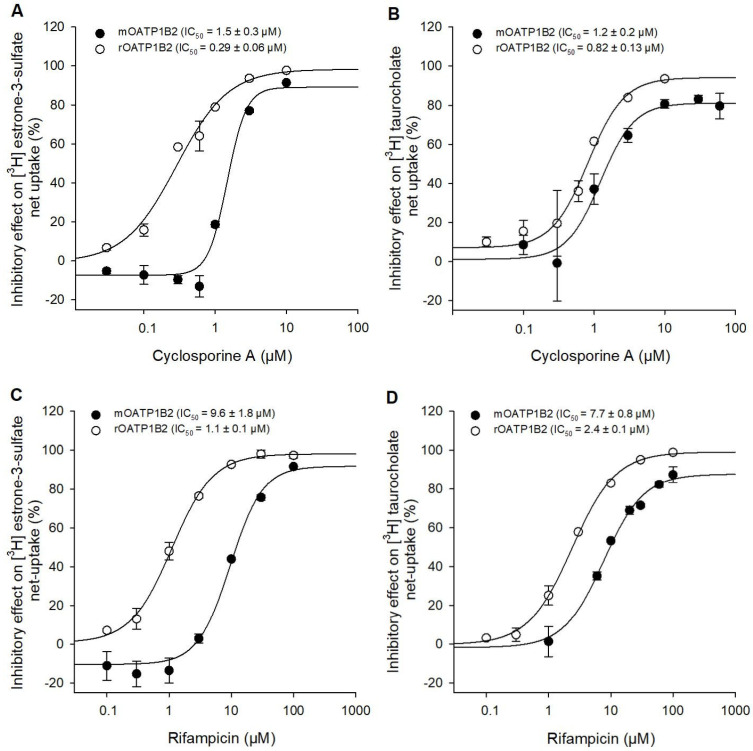
Concentration-dependent inhibition. Inhibitory effects of increasing concentrations of cyclosporine A and rifampicin on mOATP1B2 and rOATP1B2-specific uptake of 1 µM [^3^H]-E_1_S (**A**,**C**) or 1 µM [^3^H]-TCA (**B**,**D**), respectively. Data points represent the mean inhibitory effect (%) of two independent experiments ± average deviation. IC_50_ values were calculated by sigmoidal four-parameter Hill analysis using Sigma Plot version 13.0.

For a more detailed characterization of the inhibition properties, we chose the strongest inhibitor from the single-point inhibition, cyclosporine A, and the inhibitor with the largest difference between mouse and rat, the antibiotic rifampicin. For these two drugs, concentration-dependent inhibition studies were conducted (Figure 4), again with the two substrates [^3^H]-estrone-3-sulfate (Figure 4A,C) and [^3^H]-taurocholic acid (Figure 4B,D), respectively.

Uptake of 1 µM [^3^H]-estrone-3-sulfate was inhibited with increasing concentrations (0.03–10 µM) of cyclosporine A resulting in IC_50_ values of 1.5 ± 0.3 µM for mOATP1B2 and 0.29 ± 0.06 µM for rOATP1B2, which means a 5.2-fold stronger inhibition of the rOATP1B2 than of the mOATP1B2 under the same experimental conditions. However, the inhibition of 1 µM [^3^H]-TCA uptake by cyclosporine A showed, with IC_50_ values of 1.2 ± 0.2 µM for mOATP1B2 and 0.82 ± 0.13 µM for rOATP1B2, no relevant differences. The strongest difference was observed when considering the inhibition of [^3^H]-E_1_S by rifampicin. Here, an IC_50_ of 9.6 ± 1.8 µM was calculated for mOATP1B2 and 1.1 ± 0.1 µM for rOATP1B2, corresponding to an 8.7-fold difference between the two species. Again, the differences for [^3^H]-taurocholic acid uptake are smaller, 7.7 ± 0.8 µM for mOATP1B2 and 2.4 ± 0.1 µM for rOATP1B2, which means a 3.2-fold difference. In conclusion, despite these differences, both inhibitors, cyclosporine A and rifampicin, are suitable inhibitors with low IC_50_ values for mouse and rat OATP1B2.

### 2.4. Comparison of Transport Activities of Rodent OATP1B2 with the Human Orthologues hOATP1B1 and hOATP1B3

**Figure 5:**
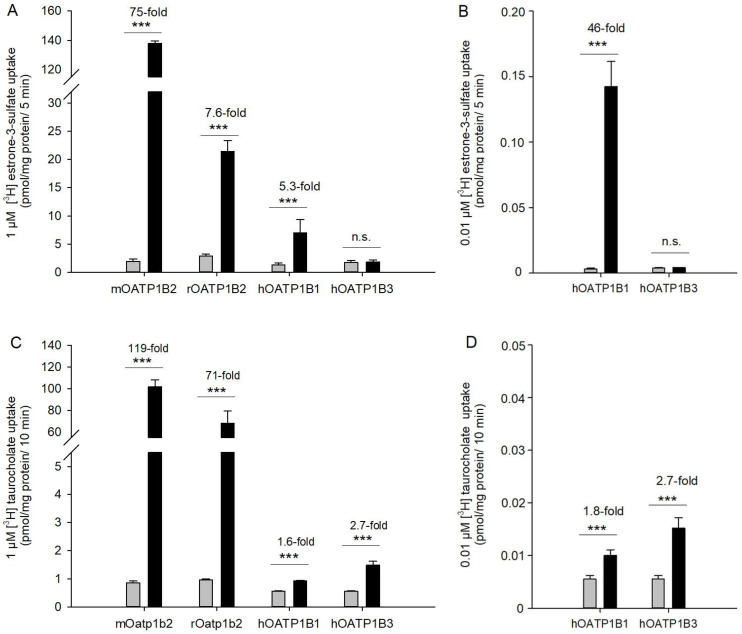
Comparison of transport activities of rodent OATP1B2 with the human orthologues hOATP1B1 and hOATP1B3. Uptake of (**A**) 1 µM [^3^H]-E_1_S or (**C**) 1 µM [^3^H]-TCA (each containing 10 nM radiolabeled substrate) in rodent and human orthologues (black bars) and vector-transfected control cells (grey bars), and uptake of (**B**) 0.01 µM [^3^H]-E_1_S or (**D**) 0.01 µM [^3^H]-TCA in human OATP1B1 and OATP1B3. Data are presented as mean ± average deviation from two independent experiments (*n* = 6). n.s. not significant,*** *p* < 0.001 Student’s *t*-test.

In drug development, it is important to ascertain the extent to which data derived from rodent livers can be extrapolated to the human liver. In this context, the functional properties of rOATP1B2 and mOATP1B2 are of particular interest, especially when compared with the orthologous human transporters. In order to gain further insight into the species-specific variations, an examination of the substrate uptake of human and rodent organic anion-transporting polypeptides was conducted. A comparison of the estrone-3-sulfate uptake of the rodent OATP1B2 with the human OATP1B1 (see Figure 5A,B) at 1 µM revealed that the cellular accumulation in human OATP1B1 was lower than in rodents. However, the uptake of 0.01 µM [^3^H]-E_1_S showed a 46-fold uptake compared to the control cells, representing a high affinity of hOATP1B1 for estrone-3-sulfate. Conversely, hOATP1B3 demonstrated no significant uptake of estrone-3-sulfate at either concentration.

Taurocholate accumulation in mOATP1B2- and rOATP1B2-transfected HEK293 cells was found to be more than 100- or 70-fold compared to vector-transfected control cells (Figure 5C). While TCA is an appropriate substrate for the rodent OATP1B2, it is poorly taken up by hOATP1B1 and hOATP1B3. The uptake ratio of TCA in hOATP1B1 cells was less than 2-fold; thus, no further experiments were conducted. Human OATP1B3 exhibited slightly higher affinity for TCA than hOATP1B1, with a 2.7-fold uptake compared to controls (Figure 5D). Consequently, a K_m_ value of 21 ± 8 µM could be ascertained (Table 1).

### 2.5. Interaction of Drugs with Human OATP1B1 and OATP1B3 Compared to Rodent OATP1B2

**Figure 6:**
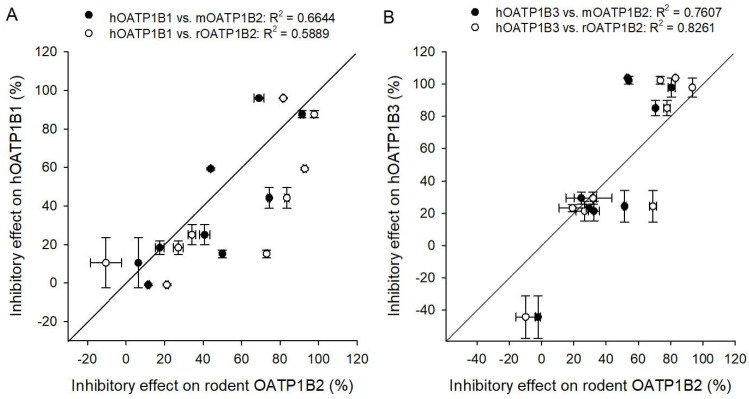
Correlation of human and rodent inhibitory effects. Scatterplot analysis correlating inhibitory effects of nine different drugs on (**A**) hOATP1B1- versus rodent OATP1B2-mediated E_1_S uptake and (**B**) hOATP1B3- versus rodent OATP1B2-mediated TCA uptake. Data are presented as mean ± average deviation from two independent experiments (*n* = 6). Lines indicate a perfect correlation.

With the aim of comparing the inhibition patterns of the rodent OATP1B2 with those of the human orthologues, an interaction study of hOATP1B1-mediated E_1_S and hOATP1B3-mediated TCA uptake was conducted using the same drugs as for the rodent OATP1B2. A regression analysis of inhibitory effects revealed in correlation coefficients (R^2^) for hOATP1B1 and mOATP1B2 of 0.6644 and for hOATP1B1 and rOATP1B2 of 0.5889 (Figure 6A).

Some differential species-dependent effects were observed. In detail, the inhibition pattern demonstrated that rosuvastatin exhibited the most substantial discrepancy, with inhibitory effects of 50% or more for mouse and rat OATP1B2, while the inhibitory effect on hOATP1B1-mediated E_1_S uptake was only 16% (see Table 1). A further difference was observed in the inhibitory effect of ritonavir, which exhibited an effect of 44% on hOATP1B1 but more than 70% on rodent OATP1B2.

A comparative analysis of inhibitory effects on hOATP1B3-mediated TCA uptake in relation to rodent OATP1B2 data generated correlation coefficients (R^2^) of 0.7607 for hOATP1B3 and mOATP1B2 and 0.8261 for hOATP1B3 and rOATP1B2 (Figure 6B). These R^2^ values indicate a higher correlation than the values obtained for hOATP1B1. But once more, rosuvastatin showed the most substantial difference, with an inhibitory effect on hOATP1B3-mediated TCA uptake of only 24% (see Table 1). No difference was observed in the inhibitory effect of ritonavir. Both human OATPs were completely inhibited by 10 µM of the ASBT-inhibitor odevixibat, while the inhibition of rodent OATP1B2 demonstrated lower inhibitory effects ranging from 54 to 82%.

## 3. Discussion

Murine OATP1B2 is highly expressed in the liver [2] similar to its human (OATP1B1/OATP1B3) and rat (rOATP1B2) orthologues, respectively. In humans, OATP1B1 and OATP1B3 are known to mediate the uptake of a large number of drugs and are considered clinically relevant transporters for hepatic uptake due to their significant role in in drug pharmacokinetics and drug–drug interactions. However, preclinical drug development typically relies initially on animal models, especially rodents, to predict pharmacokinetic behavior of new molecular entities. Given this, a thorough understanding of interspecies differences is essential to evaluate the translational value of animal data for human applications. In vitro experiments can be used to show how the functions of individual transport proteins differ from one species to another.

In this study, we present a comprehensive functional characterization of murine OATP1B2, for which only limited data were available. Systematically, we compared the transport characteristics of mOATP1B2 with those of rat OATP1B2 under identical experimental conditions. Additionally, we included data on the human orthologues OATP1B1 and OATP1B3 (Table 1) to assess the extent to which rodent-derived data can be extrapolated to human transporter function.

We report, for the first time, kinetic parameters for the uptake of estrone-3-sulfate (E_1_S) and taurocholic acid (TCA) by mOATP1B2 overexpressing HEK239 cells. The results revealed that the affinity of mOATP1B2 for both E_1_S and TCA was moderately lower than that of rOATP1B2 (2.7-fold and 4.6-fold, respectively). For both rodent transporters, TCA showed a slightly higher affinity compared to E_1_S. Interestingly, the human orthologues exhibited distinctly divergent substrate preferences, with OATP1B1 showing only marginal transport of TCA (<2-fold over control cells) but exhibiting highly efficient E_1_S uptake. Conversely, OATP1B3 mediated no transport of E_1_S but slight uptake of TCA (2-3-fold over control cells). In the literature, a Michaelis–Menten constant (K_m_) for the affinity of hOATP1B1 to TCA is not available, which leads to the conclusion that hOATP1B1 does not transport TCA to a relevant extent. An analysis of the substrate E_1_S reveals that mOATP1B2 and rOATP1B2 facilitate E_1_S transport with a comparatively low binding affinity. In contrast, hOATP1B1 exhibits a remarkably high affinity for E_1_S, with a K_m_ of 0.25 ± 0.04 µM, as reported in a previous study [14]. This indicates a substantially higher binding affinity of hOATP1B1 for E_1_S (968-fold and 360-fold) in comparison to mOATP1B2 (242 ± 23 µM) and rOATP1B2 (90 ± 11 µM), respectively. In the present study, hOATP1B3 demonstrated no substantial uptake of E_1_S, a finding consistent with the observations reported by others [15]. This outcome contrasts with the findings reported by Gui et al. (2008), who documented a K_m_ value for OATP1B3-mediated E_1_S of 58 ± 20 µM in CHO cells, however without including uptake ratios [16]. Possible explanations for such discrepancies could be differential experimental conditions like uptake time, transport buffer composition, or differential cell lines.

For hOATP1B3, Abe et al. (2001) published a K_m_ for TCA from OATP1B3-expressing oocytes of 5.8 ± 1.2 µM, but no data are available for overexpressing cell lines [17]. Therefore, we conducted hOATP1B3-mediated concentration-dependent TCA uptake, which resulted in a K_m_ of 21 ± 8 µM (see Table 1). Minor transport activity of 1 µM TCA by human OATP1B1 was observed in our HEK293 overexpression system (1.6-fold compared to vector-transfected control HEK293 cells). In humans, the transport of TCA and other conjugated bile acids into hepatocytes is primarily facilitated by the Na+-taurocholate cotransporting polypeptide (NTCP) [18,19]. For human NTCP, rodent orthologues (mouse and rat NTCP) have also been identified, sharing a sequence identity of >73% with their human counterpart [18]. In the context of species-dependent differences, Hussner et al. (2021) discovered functional differences between the orthologues hOATP2B1 and rOATP2B1 [20]. For instance, estrone-3-sulfate was transported by hOATP2B1 but not by the rodent orthologues.

Subsequently, we evaluated the inhibition properties of mOATP1B2 and rOATP1B2 by testing nine pharmacologically diverse drugs, aiming to identify a potent mOATP1B2 inhibitor and to uncover species- and substrate-specific differences. At a concentration of 10 µM, five compounds showed 50% and more inhibition of mOATP1B2-mediated substrate uptake: cyclosporine A, ritonavir, odevixibat, rosuvastatin, and rifampicin. A correlation analysis of inhibitory effects yielded strong concordance between mOATP1B2 and rOATP1B2 inhibition (R^2^ = 0.78 for E_1_S and R^2^ = 0.91 for TCA), indicating a high degree of similarity. However, individual differences were observed, most notably for rifampicin, which exhibited a >30% difference in inhibition. Based on these results, we selected rifampicin and cyclosporine A for further characterization. The immunosuppressant cyclosporine A inhibits many human uptake transporters, including hOATP1B1 and hOATP1B3 (see Table 1), as well as several efflux transporters such as MDR1, MRP2, MRP3, and BCRP [21]. Fricker et al. (1996) additionally identified cyclosporine A as a substrate of MDR1, with a reported K_m_ value of 3.8 µM [22]. In our study, cyclosporine A inhibited mOATP1B2- and rOATP1B2-mediated substrate uptake with IC_50_ values around 1 µM. An analysis of the inhibition data for hOATP1B1 and hOATP1B3 (see Table 1) with 10 µM cyclosporine A reveals that they exhibit an inhibitory effect of 90%, which is consistent with the range observed for the rodent OATP1B2. Furthermore, Karlgren et al. (2012) reported IC_50_ values for hOATP1B1 and hOATP1B3 of 1.4 and 1.3 µM, respectively [21].

Rifampicin demonstrated IC_50_ values for rOATP1B2 of 1.1 µM (E_1_S) and 2.4 µM (TCA). In contrast, mOATP1B2 showed 3- to 9-fold higher IC_50_ values (9.6 µM for E_1_S and 7.7 µM for TCA), suggesting a species-specific difference in rifampicin sensitivity. Interestingly, the inhibitory effects of 10 µM rifampicin on hOATP1B1 and hOATP1B3-mediated substrate uptake were 59% and 104% (see Table 1) and also divergent. However, it is important to consider that different substrates were used. The regression analysis demonstrated a high degree of correlation among the species; however, individual compounds exhibited divergent substrate inhibition. Similar deviations are also observed between the two human transporters hOATP1B1 and hOATP1B3. Karlgren et al. (2012) showed an overlap of inhibitors between these two transporters of 42 of 82 but also found 27 specific inhibitors for hOAPT1B1 and 3 specific inhibitors for hOATP1B3 [21].

Ritonavir demonstrated consistent inhibition, exhibiting inhibitory effects ranging from 71% to 85% for mouse and rat OATP1B2 and hOATP1B3, but its inhibitory effect on hOATP1B1-mediated E_1_S uptake was slightly lower, with an observed value of 44%. Rosuvastatin was described as a potent substrate for hOATP1B1 (K_m_: 0.8 µM) and hOATP1B3 (K_m_: 14.2 µM) [13]. In the present study, the interaction of E_1_S and TCA uptake with 10 µM rosuvastatin resulted in inhibitory effects of only 16% and 24% for hOATP1B1 and hOATP1B3. In contrast, the inhibitory effects on mOATP1B2 (50% and 52%) and rOATP1B2 (73% and 69%)-mediated E_1_S and TCA uptake were stronger. Ishida et al. (2018) showed rOATP1B2-mediated uptake of rosuvastatin (K_m_: 28.1 µM) [9], but further investigations are required to prove whether rosuvastatin is also a substrate for mOATP1B2.

Notably, another potent inhibition was detected with odevixibat. For the human OATP1B1/1B3, a complete inhibition of substrate (E_1_S/TCA) uptake was observed at 10 µM odevixibat. The inhibitory effects on mOATP1B2 (69% and 54%) and rOATP1B2 (82% and 74%)-mediated substrate uptake were less pronounced. Certainly, the clinical relevance of these results remains unlikely due to the very low C_max_ value for odevixibat [23] used to treat progressive familial intrahepatic cholestasis by inhibiting the ileal sodium/bile acid cotransporter (ASBT) and thereby reducing the reabsorption of bile acids in the distal ileum [24].

In this study, clotrimazole did not inhibit any of the estimated OATPs at a concentration of 10 µM. However, previous research has characterized clotrimazole as an inhibitor of hOATP1B1, with an IC_50_ of 9 µM [16]. In addition, the three substances ketoconazole, olmesartan, and amprenavir showed less than 50% percent inhibition for all the transporters that were examined. The published IC_50_ values for amprenavir are 16.8 µM for OATP1B1 [21] and 19.1 µM for OATP1B3 [25], which is consistent with an inhibition less than 50% in this study. Olmesartan was described as a substrate of hOATP1B1, hOATP1B3 [12], and rOATP1B2 [9]. In this study, we observed inhibitory effects ranging from 18% to 32% for both rodent OATPs as well as human OATPs, indicating competitive inhibition. Nevertheless, further investigation is necessary to verify whether olmesartan functions as a substrate for mOATP1B2. Ketoconazole was expected to demonstrate a stronger inhibitory effect, given an IC_50_ value of 1.8 µM for hOATP1B1 [26]. However, the use of estradiol-17β-glucuronide as a substrate in that study could potentially explain the observed discrepancies.

## 4. Conclusions

In conclusion, on the one hand, the rodent transporters mOATP1B2 and rOATP1B2 appear to be suitable models for drug inhibition studies and provide a good basis for extrapolating data to human orthologous transporter systems. However, the observed differences in substrate affinity and transport capacity emphasize the need for caution when extrapolating rodent data to human systems. Taken together, the disparity in substrate binding affinity between mOATP1B2 orthologues suggests the presence of relevant differences in the substrate binding sites between rodent and human transporters.

These findings underscore the importance of comparative in vitro studies across species to better understand transporter-mediated drug disposition and to improve the predictive accuracy of preclinical models.

## 5. Materials and Methods

### 5.1. Material

[^3^H]-estrone-3-sulfate [6,7-^3^H(N)] ammonium salt ([^3^H]-E_1_S, ART0821, specific activity 40 Ci/mmol) and [^3^H]-taurocholic acid ([^3^H]-TCA, ART1368 specific activity 20 Ci/mmol) were obtained from American Radiolabeled Chemicals (Saint Louis, MO, USA). FBS premium and DMEM-high glucose medium were obtained from Biowest (Nuaillé, France); hygromycin B, Bradford solution, and scintillation cocktail Rotiszint^®^eco plus were obtained from Carl Roth (Karlsruhe, Germany). Unless otherwise noted, all compounds (drugs) used in the study as well as Hank’s balanced salt solution (138 mM NaCl, 5.33 mM KCl, 1.26 mM CaCl_2_, 0.41 mM MgSO_4_, 0.44 mM KH_2_PO_4_, 4.0 mM NaHCO_3_, 0.3 mM Na_2_HPO_4_, and 5.6 mM Glucose), HEPES, trypsin, penicillin/streptomycin, and PBS were obtained from Sigma-Aldrich (Munich, Germany).

### 5.2. Methods

#### 5.2.1. Transfection and Cell Culture

For stable transfection, Flp-In™ T-REx™ 293 Cell Line and pcDNA™5/FRT/TO (Thermo Fisher Scientific/Invitrogen, Darmstadt, Germany) were used. The pcDNA5/FRT/TO::mOATP1B2 (NCBI database www.ncbi.nlm.nih.gov, accession number NM_020495.2, codon optimized) and pcDNA5/FRT/TO::rOATP1B2 (NCBI database www.ncbi.nlm.nih.gov, accession number NM_031650.3) constructs were purchased from BioCat (Heidelberg, Germany). The human embryonic kidney cells (Flp-In™ T-REx™ 293 Cell Line, parental cell line ATCC Number CRL-1573) were transfected by incubating 0.35 µg pcDNA5/FRT/TO vectors (containing the respective cDNA) and 3 µg pOG44 (ratio 1:9) as well as 10 µL Lipofectamine^TM^ 2000 (Thermo Fisher Scientific/Invitrogen, Darmstadt, Germany) in 500 µL serum-free DMEM transfection medium for 5 h at 37 °C. Then, the medium was changed to Dulbecco’s modified Eagle’s medium (DMEM, high glucose) supplemented with 10% fetal bovine serum and 1% penicillin (10.000 Units/mL)/streptomycin (10 mg/mL). The next day, the cells were transferred to a 10 mm cell culture dish and the selection agent hygromycin B (175 µg/mL) was added to the medium after 5 h. Three weeks later, the single cell foci were numbered, and each was transferred into a single well in a 24-well plate. HEK-293 cells stably expressing mOATP1B2 or empty vector control cells were selected in the presence of hygromycin B (175 µg/mL). Well-growing clones were expanded, and gene expression was analyzed by real-time PCR. Then, the transporter-transfected and vector-transfected HEK293 cells were grown on 100 mm or 150 mm diameter cell culture dishes at 37 °C in a humidified 5% CO2 [*v*/*v*] atmosphere. The human OATP1B1 and OATP1B3 stably transfected HEK-293 cells were generated as previously described in Marada et al. (2015) [14].

All HEK-293 cell lines (mOATP1B2-, rOATP1B2, hOATP1B1, hOATP1B3-, and the vector-HEK cells) were tested for mycoplasma routinely using the Mycoplasma Detection Kit (Venor^®^GeM, Minerva biolabs, Berlin, Germany) for conventional PCR and used if free (tested negative) from mycoplasma.

#### 5.2.2. Transporter-Mediated Uptake of Radiolabeled Substrates

To perform transporter-mediated uptake, both stably transfected cells and control cells (seeding density 2 × 10^5^) were cultured for three days in 24-well plates (coated with 0.1 mg/mL poly-D-lysine hydrobromide). Then, 24 h before the start of the transport experiment, the gene expression of mOapt1b2 and rOATP1B2 was induced by 1 µg/mL tetracycline. At the beginning of each experiment, the cells were washed three times with pre-warmed 0.5 mL HBSS incubation buffer (supplemented with 20 mM HEPES, pH 7.4) and equilibrated for at least 20 min as described previously [27]. After aspirating the incubation buffer, the uptake process was started by adding 200 µL HBSS incubation buffer containing radio-labeled and unlabeled substances to each well and incubated at 37 °C for an appropriate time. The uptake was stopped by aspirating the reaction mixture and washing the cells 3 times with 0.4 mL ice-cold PBS buffer. After cell lysis with 0.6 mL of 1N NaOH overnight, the whole content of each well (0.6 mL) was transferred to a 6 mL scintillation vial (Sarstedt) containing 2.5 mL scintillation liquid (Rotiszint^®^eco plus, Carl Roth). The ^3^H content was measured in a scintillation counter (Perkin Elmer TRICarb 2810 or HIDEX 600SL).

The uptake experiments in stably transfected HEK293 cells were carried out with [^3^H]-E_1_S and [^3^H]-TCA as the probe substrates. The linearity of the mOATP1B2- and rOATP1B2-mediated E_1_S and TCA uptake was determined by time-series experiments with eight time intervals (1, 2, 3, 4, 5, 10, 20, and 30 min) at a concentration of 1 µM E_1_S and TCA (containing each 0.01 µM radiolabeled compound). The affinity of mOATP1B2 and rOATP1B2 for the substrates E_1_S and TCA, respectively, were determined as K_m_ values (Michaelis–Menten constant) using increasing substrate concentrations. Therefore, mOATP1B2 or rOATP1B2- and the corresponding vector-HEK cells were incubated for 1 min with E_1_S or TCA concentrations between 0.01 and 500 µM (each concentration contained 0.01 µM radiolabeled compound). Furthermore, the affinity of the human OATP1B3 for TCA was determined as K_m_ under the following conditions: OATP1B3-HEK and vector-HEK cells were incubated for 10 min with increasing TCA concentrations: (0.01, 0.1, 0.5, 1, 5, 10, 50, and 100; each concentration contained 0.01 µM radiolabeled compound).

For the comparison of substrate uptake in human and rodent orthologues, mOATP1B2-, rOATP1B2-, hOATP1B1-, and hOATP1B3-mediated [^3^H]-E_1_S and [^3^H]-TCA uptake were examined at 1 µM substrate concentration. Incubation with E_1_S was carried out for 5 min and for TCA for 10 min. To verify the results for the human transporters, both substrates were tested at a concentration of 0.01 µM. All experiments were conducted at least on two separate days as triplicates.

#### 5.2.3. Inhibition Experiments

Inhibition experiments with mOATP1B2 and rOATP2B1 were carried out in triplicate as cis-inhibition. The uptake of the ^3^H-labeled probe substrates E_1_S or TCA (1 µM, containing 10 nM radiolabeled [^3^H]-E_1_S or [^3^H]-TCA) was measured after a one-minute incubation period in the absence or presence of 10 µM of the respective drugs.

For IC_50_ determination, moatp1b2- or roatp1b2-mediated E_1_S or TCA uptake was cis-inhibited by increasing concentrations of the respective inhibitors. The cells were incubated for 1 min with 1 µM E_1_S or TCA and seven concentrations of the inhibitor. The E_1_S or TCA solutions contained 0.01 µM labeled ^3^H-substrate, respectively.

For inhibition experiments with hOATP1B1 and hOATP1B3, cis-inhibition was carried out in triplicate by measuring the uptake of the ^3^H-labeled probe substrate 0.01 µM [^3^H]-E_1_S for hOATP1B1 (1 min) or 1 µM TCA (containing 0.01 µM [^3^H]-TCA) for hOATP1B3 (10 min) in the absence and presence of 10 µM of the respective drugs.

Inhibitory effects were calculated as a percentage of the net-uptake rates. All experiments were conducted at least on two separate days as triplicates.

#### 5.2.4. Determination of Protein Amount

The amount of cellular protein was quantified by the Bradford method [28]. The cell monolayers in 24-well plates were washed three times with incubation buffer and stored at −20 °C. For protein determination, the plates were thawed, and each well was incubated for lysis 30 to 60 min in 100 µL 1X lysis buffer (5X lysis buffer; Promega; diluted 1:4 [*v*/*v*] in ddH_2_O). The cell lysate was filled with ddH2O to 2 mL per well and mixed thoroughly. The measurement was performed in 96-well plates (Sarstedt; flat bottom), in duplicate. BSA (working solution: 1 mg/mL) was used as standard for a calibration curve ranging from 50 to 300 µg/mL BSA. A total of 20 µL of BSA-standard or 20 µL sample (diluted 1:1 [*v*/*v*] with ddH_2_O) was mixed with 200 µL 1X Bradford-reagent (diluted from 5X stock reagent; Carl Roth) per well. After 5 to 10 min incubation at room temperature, absorption was measured at 590 nm (Berthold Technologies TriStar2 LB942 spectrophotometer).

#### 5.2.5. Data Analysis

The absolute amount (pmol) of the radio-labeled probe substrate uptake was calculated and related to the determined protein values as well as the incubation time. The transporter-mediated uptake rate (net uptake) was obtained by subtracting the uptake rate in control (vector only)-transfected HEK293 cells from the uptake rate in transporter-transfected cells. To calculate the K_m_ of the respective substrate, the transporter-mediated net uptake (pmol/mg protein/min) was plotted against the substrate concentrations in SigmaPlot 13. Weighted nonlinear regression was used for curve fitting (ligand binding with one site saturation).

The inhibitory effect I (%) was calculated as I (%) =100 − (V_with inhibitor_ × 100/V_w/o inhibitor_), and for the IC_50_ calculation, the inhibitory effect was plotted against the inhibitor concentrations and fitted using the four-parameter Hill equation. The means ± average deviation (AD, for *N* = 2) were calculated from two experiments on two different days. The significance as a *p*-value was calculated using Student’s *t*-test in Microsoft Excel.

## Figures and Tables

**Table 1:** Overview of mouse OATP1B2 and rat OATP1B2 parameters compared to human OATP1B1 and OATP1B3.

	mOATP1B2		rOATP1B2		hOATP1B1		hOATP1B3	
Protein length (amino acids)	689		687		691		702	
Identity to mOATP1B2 (%)	100		81.46		64.23		66.18	
Probe substrates	E_1_S	TCA	E_1_S	TCA	E_1_S	TCA	E_1_S	TCA
K_m_ (µM)	242 ± 23	73 ± 11	90 ± 11	16 ± 3	0.25 ± 0.04 [14]	Uptake < 2	no substrate	21 ± 8
	Inhibitory effects at 10 µM of the drug (%)							
Cyclosporine A	91	81	98	94	88	-	-	98
Rifampicin	44	53	93	83	59	-	-	104
Ritonavir	74	71	84	78	44	-	-	85
Odevixibat	69	54	82	74	96	-	-	102
Rosuvastatin	50	52	73	69	16	-	-	24
Ketoconazole	41	29	34	19	25	-	-	23
Olmesartan	18	25	27	32	18	-	-	29
Amprenavir	12	32	21	27	−1	-	-	21
Clotrimazole	6	−2	−10	−10	10	-	-	−44

## Data Availability

The raw data supporting the conclusions of this article will be made available by the authors on request.
